# Comparison of vascular smooth muscle cells in canine great vessels

**DOI:** 10.1186/1746-6148-9-54

**Published:** 2013-03-25

**Authors:** Noriko Isayama, Goki Matsumura, Kenji Yamazaki

**Affiliations:** 1 Cardiovascular Surgery, The Heart Institute of Japan, Tokyo Women’s Medical University, 8-1 Kawada-cho, Shinjuku-ku, Tokyo 162-8666, Japan

**Keywords:** Canine, Smooth muscle cells, Vessel, Histology

## Abstract

**Background:**

Elucidating the histological characteristics of normal vascular smooth muscle cells (VSMCs) is important for understanding mechanisms of development, disease etiology and the remodeling and/or regeneration process of the vessel. However, knowledge regarding VSMCs is focused primarily on the artery. Although the characteristics of each great vessel are documented, few studies have examined VSMCs in parallel within each great vessel. The present study focused on comparing characteristics of canine VSMCs within the aorta (Ao), branch pulmonary artery (bPA), main pulmonary artery (mPA) and inferior vena cava (IVC), simultaneously.

**Results:**

Western blot and immunohistochemistry were used to determine VSMC protein content for alpha smooth muscle actin (ASMA), calponin, myosin heavy chain (MHC) and its isozyme SM2, and non-muscle myosin heavy chain B (SMemb). Thickness and ratio of the VSMC layer were also measured. Expression levels of ASMA, calponin and SM2 significantly differed between vessels, except between mPA and either bPA, Ao and IVC vessels. Expression levels of MHC were significantly different in all vessels, whilst expression of SMemb was significantly different in the Ao compared with either bPA and mPA vessels. All vessels were significantly different with respect to total wall and VSMC layer thickness. The ratio between VSMC layer and total wall thickness was significantly different for each vessel, except between bPA and mPA vessels. Histological analysis of the IVC revealed that the VSMC layer does not line evenly and continuously through the long axis or transverse sections. With respect to the pulmonary artery, calponin was expressed to a greater extent in the mPA compared with the bPA (P < 0.01*). In contrast, MHC and SM2 were expressed to a greater extent in the bPA compared with the mPA (P < 0.01*). Differences in VSMC distribution indicate structural differences in the proximal and distal pulmonary artery bifurcation.

**Conclusion:**

Our results show that the VSMC expression pattern in each great vessel is unique and suggestive of the developmental differences between great vessels. We believe this study provides basic data for the pathology, etiology and regenerative capability of the vessels.

## Background

Various antibodies specific for vascular smooth muscle cells (VSMCs) exist, although their application in recent investigations of normal vessels has been limited. Elucidation of the histological distribution of normal VSMCs is important for understanding mechanisms of development, causes of hypertension, occurrence of leiomyoma, remodeling following vascular injury and regenerative medicine approaches.

Vascular walls comprise an endothelial layer, composed of a tunica intima, a layer of smooth muscle cells; tunica media; and tunica externa, outer connective tissue layer. However, the volume and distribution of these layers differs remarkably with regard to the role of the vessels. It is well-documented that arteries and veins display distinct histological differences. The arterial walls consist of a substantial smooth muscle layer, to respond to acute hemodynamic alteration through either constriction or dilatation. In contrast, the venous wall has a thinner smooth muscle layer with an undefined border, to accommodate blood volume changes. The architecture of the vena cava is similar to that of the arteries, although the smooth muscle layer is still much thinner [1].

The main smooth muscle contractile proteins are actin and myosin. The expression level of these proteins has been reported to vary in different smooth muscle types. Smooth muscle actin exists in several isoforms, α, β and γ. The β-isoform generates cytoskeletal actin, whereas α and γ generate the contractile apparatus. Actin-binding proteins, such as tropomyosin, caldesmon and calponin, play a role in the thin filament-based regulation of smooth muscle contractility [2].

Calponin (34-kDa protein) interacts with F-actin and tropomyosin in a Ca^2+^-independent manner and with calmodulin in a Ca^2+^-dependent manner. It is present in smooth muscle at the same molar concentration as tropomyosin [2]. The myosin molecule is a hexametric motor-enzymic protein, consisting of two myosin heavy chains (MHC) of approximately 200 kDa and four myosin light chains (MLC) of approximately 16–27 kDa. Within smooth muscle cells, MHCs exists in two isoforms; SM1 (204 kDa) and SM2 (200 kDa) [3-5]. Two non-muscle MHCs, NMHC-A and NMHC-B, are also present, but are not unique to smooth muscle cells.

SM1 and SM2 genes are expressed exclusively in smooth muscle-containing tissues throughout development. SM1 is constitutively expressed at all developmental stages, whereas SM2 appears only after birth [6]. NMHC-B is identical to SMemb which is expressed in the embryonic aorta and in neointimal lesions [7,8]. During development, SMemb may be down regulated and replaced with adult smooth muscle [9] and be re-expressed in the proliferating SMC neointima [10].

Furthermore, information on vascular smooth muscle cell content has mainly focused on the artery. Few reports compare the differences in smooth muscle cells between arteries and veins. It has been reported that in canine mesenteric arteries and veins, the content of actin, MHC, MLC and calponin are similar [11]. However, the expression pattern of MHC genes is significantly different in the inferior vena cava compared with the aorta and pulmonary artery of fetal and adult rabbits [12,13].

Understanding the basic foundations of a native vasculature is necessary for elucidating the development of pathophysiology and regenerative medicine. Recently, there has been great interest in regenerative medicine studies. We have previously reported long-term results of tissue-engineering vasculature in a canine vena cava model [14]. This vasculature was well remodeled into a native-like vessel with no calcification, stenosis, or rupture. Similar studies have been carried out in the pulmonary artery [15]. Nevertheless, it is difficult to measure the regenerate level of these remodeled vasculature studies because of a lack of information regarding native vessels.

The present study investigated canine great vessels adjacent to the heart, including the aorta, pulmonary artery and vena cava, to identify differences in expression levels of the major contractile and thin filament-binding proteins and thereby fill a deficit of vascular smooth muscle cell research.

## Results

### Western blot analysis

**Figure 1:**
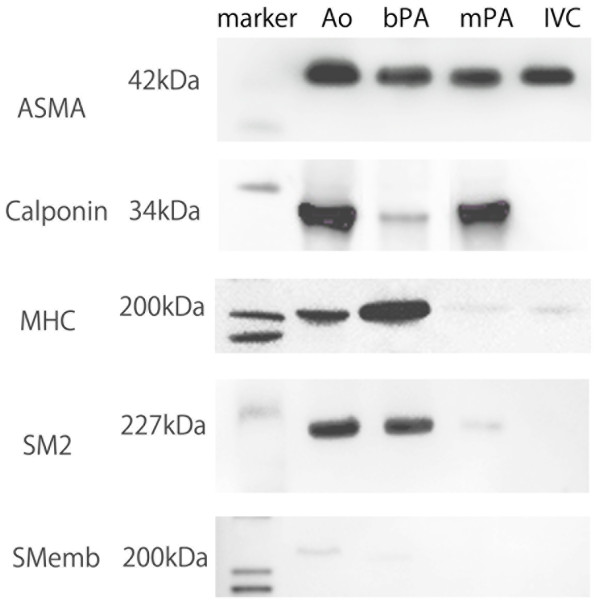
**Western blot analysis of Ao, bPA, mPA and IVC.** Total protein from Ao, bPA, mPA and IVC tissues were subjected to western blot analysis for measurement of ASMA, calponin, MHC, SM2 and SMemb expression. Ao; ascending aorta, bPA; branch pulmonary artery, mPA; main pulmonary artery, IVC; inferior vena cava.

**Figure 2:**
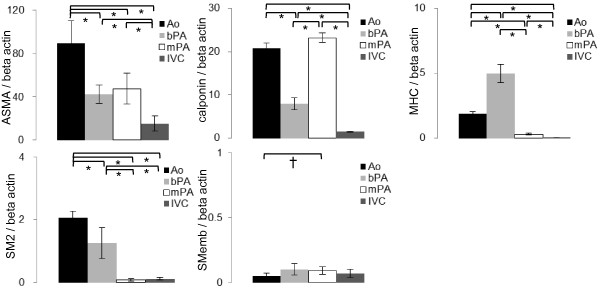
**Bar graphs showing protein densitometry of ASMA, calponin, MHC, SM2 and SMemb.** All samples are normalized to β actin protein in VSMCs of the Ao, bPA, mPA and IVC. Ao; ascending aorta, bPA; branch pulmonary artery, mPA; main pulmonary artery, IVC; inferior vena cava. * Significant at *p* <0.01, † significant at *p* <0.05. Error bar represents standard error of the mean.

All antibodies reacted positively to their respective canine VSMC proteins (Figure 1). Representative data for the contractile and thin filament-binding proteins are shown in Figure 2. The ratios of ASMA to β actin in the Ao, bPA, mPA and IVC were 89.17 ± 8.61, 42.15 ± 3.51, 47.26 ± 5.79 and 15.08 ± 2.85, respectively. Significant differences were observed in all vessels (*p* < 0.01), except in bPA vs. mPA (*p* = 0.70). The ratios of calponin to β actin in the Ao, bPA, mPA and IVC were 20.8 ± 1.14, 7.94 ±1.35, 23.2 ± 1.15 and 1.50 ± 0.18, respectively. Significant differences were observed in all vessels (*p* <0.01), except in the Ao vs. mPA (*p* =0.18). The ratios of MHC to β actin in the Ao, bPA, mPA and IVC were 1.88 ± 0.17, 4.96 ± 0.70, 0.31 ± 0.05 and 0.06 ± 0.01, respectively. Significant differences were obtained between all vessels (*p* < 0.01). The ratios of SM2 to β actin in the Ao, bPA, mPA and IVC were 2.07 ± 0.86, 1.25 ± 0.20, 0.09 ± 0.01 and 0.12 ± 0.17, respectively. There were significant differences between all vessels (*p* < 0.01), except in mPA vs. IVC (*p* =0.24). The ratios of SMemb to β actin in the Ao, bPA, mPA and IVC were 0.05 ± 0.01, 0.10 ± 0.02, 0.09 ± 0.02 and 0.07 ± 0.01, respectively. There were significant differences between Ao vs. bPA and Ao vs. mPA (*p* < 0.05).

### Immunohistochemistry

**Figure 3:**
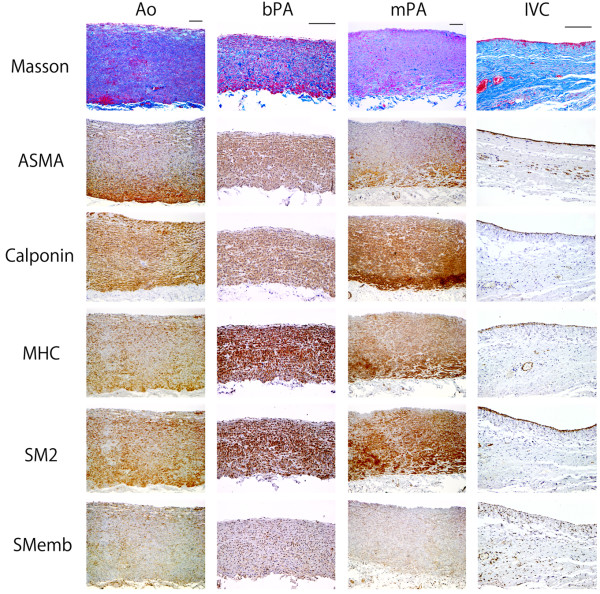
**Immunohistochemical analysis of all vessels for ASMA, calponin, MHC, SM2 and SMemb expression.** All vessels were strongly positive for cytoplasmic ASMA, calponin, MHC and SM2. Expression of SMemb was low in all vessels. Bar = 200 μm.

Positive expression of ASMA, calponin, MHC, SM2 and SMemb was strong and diffuse within the cytoplasm. Expression of SMemb was weakly positive (Figure 3).

**Figure 4:**
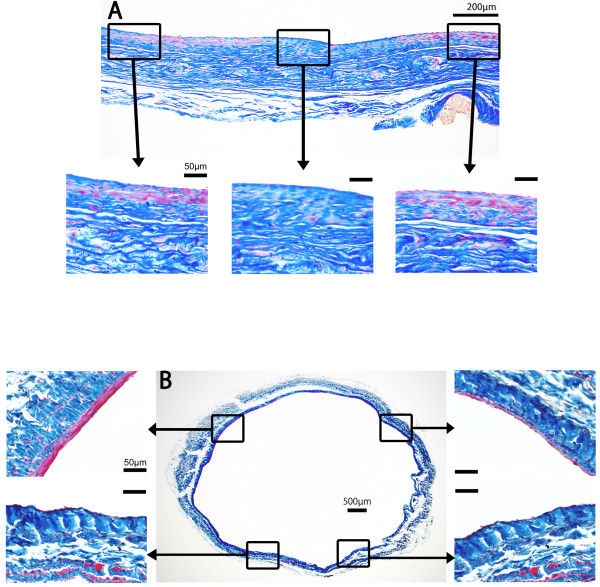
**A, Masson’s trichrome staining of longitudinally sectioned IVC sample. B**, Masson’s trichrome staining of transverse sectioned IVC sample. Each box represents higher magnifications of the arrow head-indicated images. VSMCs (red) are unevenly and non-continuously aligned in the long axis and circumference with inconsistencies between randomized sample points.

In the IVC, variations in thickness of the VSMC layer were seen after Masson’s trichrome staining. Immunohistochemistry of the IVC revealed variations in AMSA, calponin, MHC and SM2 expression. IVC tissue samples were cut transversely to evaluate variations in smooth muscle thickness (Figure 4).

### Smooth muscle thickness and ratio

**Figure 5:**
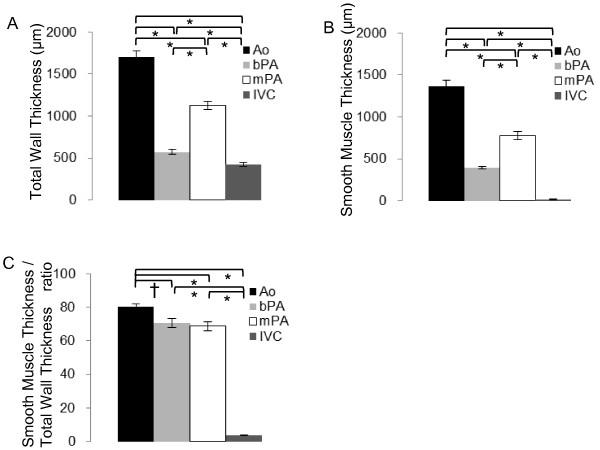
**Bar graphs showing smooth muscle thickness and ratio for each vessel. A**, total wall thickness. **B**, smooth muscle layer thickness. **C**, Ratio of smooth muscle thickness in total wall thickness. Significant differences exist between all vessels with respect to total wall thickness, smooth muscle layer and ratio. The ratio between bPA and mPA was not significantly different. Ao; Ascending aorta, bPA; branch pulmonary artery, mPA; main pulmonary artery, IVC; inferior vena cava. * Significant at *p* < 0.01, † significant at *p* <0.05. Error bar represents standard error of the mean.

**Figure 6:**
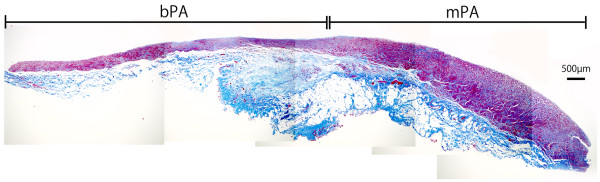
**Continuous sectioned sample of the mPA to bPA.** Total wall thickness and VSMC layer thickness were significantly different between mPA and bPA tissues, although the ratio of smooth muscle layer / wall thickness was not significantly different between mPA and bPA tissues. Bar = 500 μm.

The wall thickness of the Ao, bPA, mPA and IVC were 1700.8 ± 73.68 μm, 572.85 ± 30.32 μm, 1125.0 ± 48.10 μm and 420.14 ± 25.62 μm, respectively (Figure 5, Panel A). Significant differences were observed between all vessels. (*p* <0.01) Thickness of the tunica media of the Ao, bPA, mPA and IVC were 1368.0 ± 64.64 μm, 393.50 ± 18.50 μm, 780.79 ± 48.11 μm and 15.08 ± 1.87 μm, respectively (Figure 5, Panel B). Significant differences were measured between all vessels with respect to thickness of the tunica media (*p* <0.01). The calculated ratio of smooth muscle/wall thickness of the Ao, bPA, mPA and IVC were 80.22 ± 1.79%, 70.50 ± 15.1%, 68.80 ± 2.64% and 3.71 ± 0.42%, respectively (Figure 5, Panel C). There were significant differences between all vessels (*p* <0.05) except in bPA vs. mPA (*p* =0.72) (Figure 6).

## Discussion

Vascular smooth muscle cells (VSMCs) play an important role in vascular tone, repair, arteriogenesis, angiogenesis, and in vascular pathology. A number of studies have identified important information in relation to VSMCs regarding mechanisms of atherosclerosis, restenosis, differentiation and VSMC modulation during vascular development. However, relatively little information is available regarding the characteristics of canine VSMCs in different vessel tissues such as coronary [16], cerebral [17], renal [18], portal [19], and mesenteric [11] artery and/or vein from each expert field. In this study, we simultaneously examined VSMC characteristics in the aorta, vena cava and pulmonary arteries. This information is crucial, especially for reconstruction of the vasculature during tissue engineering approaches. Furthermore, regenerative medicine is a fast growing and high impact research area, with likely significant future developments. We previously reported the use of tissue engineered vasculature in a canine venous reconstruction model and in human clinical trials for the treatment of vascular disorders [14,20-22]. Another objective in vasculature regeneration is reconstruction of native tissue. Accordingly, it is important to compare with native vasculature to evaluate the extent of regeneration. However, few studies on normal canine great vessels, especially in the venous system and pulmonary arteries, have been carried out. This basic study was therefore important to study VSMC characteristics in normal canine great vessels and to set appropriate benchmarks for subsequent studies on tissue-engineering vasculature in animal models.

ASMA, calponin, MHC, SM2 and SMemb antibodies were used to study the characteristics of VSMCs in canine great vessels. These antibodies have been used elsewhere to show differentiation and heterogeneity of VSMCs in animal and human studies, with and without disease. Western blot analysis using these protein markers was performed in the young canine Ao, bPA, mPA and IVC vessels to examine the characteristics of VSMCs within these tissues.

ASMA is an excellent smooth muscle cell [23] marker because it is the first known protein expressed in SMC differentiation during development and is required for end-stage differentiation [24]. Calponin, a calcium regulatory protein, and MHC are exclusively expressed in SMCs [2,5,6,9,25], and stretch strain has no effect on smooth muscle expression [26]. It has been shown that antibodies against calponin and MHC do not cross-react with skeletal, cardiac non-muscle tissue myosin and myosin. MHC is therefore a reliable SMC differentiation marker since it seems highly restricted to SMCs. Other proteins, including ASMA and calponin, are also expressed in fibroblasts [23].

Quantitative western blot analysis has been previously carried out on canine mesenteric arteries and veins; however no differences were observed in the content of either actin, MHC, MLC or calponin [11]. In contrast, a difference in expression pattern of the MHC gene was reported in a separate study on rabbit great vessels [12]. We report here that VSMC in the Ao were significantly different to those in the IVC with respect to ASMA, calponin, MHC and SM2 expression. Smooth muscle thickness, total wall thickness and ratio were also significantly different between Ao and IVC tissues. These results provide further evidence that the protein content, size and location of SMC differs within vasculature tissues.

No significant difference was observed for the ratio of smooth muscle/total wall thickness between the bPA and mPA in the pulmonary arteries or in ASMA concentration between the mPA and bPA. However, calponin expression was significantly higher in mPA compared with bPA vessels. In contrast, MHC and SM2 expression was significantly higher in bPA compared with mPA vessels. These differences in distribution of the VSMC contractile and thin-filament binding proteins between mPA and bPA vessels, suggest structural differences between proximal and distal bifurcation of the pulmonary artery.

It has been reported that the pulmonary branches develop from the sixth aortic arch, while the proximal pulmonary trunk develops from cells originating in the secondary heart field [27]. This supports our observation and could explain the reasons for disparity in our analysis between the mPA and bPA vessels. Namely, the characteristic differences in VSMCs likely depend on cell origin during prenatal development.

In terms of the IVC, we first collected the tissue samples longitudinally, 1 to 4 cm adjacent to the right atrium. Histological observation revealed that the VSMC layer did not line evenly and continuously through the IVC long axis. We therefore examined the distribution of VSMCs transversely in the IVC. The thickness of the smooth muscle layer varied in the horizontal sections. Interestingly, some areas did not show any smooth muscle layer. These observations indicate that the IVC is covered with an uneven smooth muscle layer.

According to previous studies, other antibodies are known to cross-react in canine VSMCs [11]. However, we were unable to identify cross-reactivity using caldesmon, SM1 and tropomyosin-α/β antibodies in our samples. This may be due to differences in experimental conditions and the overall reactivity of the antibodies, and represents a study limitation.

## Conclusion

In conclusion, we have characterized VSMCs in canine great vessels using semi-quantitative western blot analysis and immunohistochemistry. From our results, we can determine that the VSMC expression pattern in each great vessel is regulated by size and location. We believe this current study provides the basic data for investigating vascular pathology, etiology and regenerative medicine in the aorta, pulmonary arteries and inferior vena cava.

## Methods

### Sample preparations

Tissues were obtained from 18 healthy adult female beagle dogs (age, 24 ± 7 months) (NARC Co., Tomisato, Japan). Vessel samples were excised during experimental procedures or when the dogs were sacrificed. Ascending aorta; Ao (n = 6), branch pulmonary artery; bPA (n = 6), main pulmonary artery; mPA (n = 6), and inferior vena cava; IVC (n = 6), were dissected out from sacrificed animals (n = 12) or from control animals (n = 6) during experimental procedures. Tissues were stored at −20°C for western blot analysis, or embedded in 4% paraformaldehyde for immunohistochemical study. The study was approved by the Animal Care and Use Committee of Tokyo Women’s Medical University.

### Western blot analysis

Samples were weighed and homogenized at a 1:20 (w/v) ratio of tissue to Tissue Protein Extraction Reagent (Thermo Fisher Scientific, Rockford, IL) to extract protein. The total protein content was determined using the Bradford assay. Equal amounts (15 μg) of the denatured proteins were separated in 4‒ 12% polyacrylamide gels (NuPAGER Novex^®^ Bis-Tris [Bis (2-Hydroxyethyl) amino-Tris (Hydroxymethyl) methane-HCl] Midi Gels; Invitrogen, Carlsbad, CA) and transferred to polyvinylidene difluoride (PVDF) membranes using an iBLOT dry blotting system (Invitrogen). The following antibodies were used: ASMA (1:1000; Dako, Glostrup, Denmark), calponin (1:4000; Sigma, St Louis, MO, USA), MHC (1:1000; Sigma, St Louis, MO, USA), SM2 (1:1000; Abcam, Cambridge, MA, USA), and SMemb (1:1000; Yamasa, Tokyo, Japan). β actin (1:1000; Abcam, Cambridge, MA, USA) was used as an internal control. A WesternBreeze^®^ Chemiluminescent Immunodetection Kit (Invitrogen) and BenchPro4100™ system (Invitrogen) were used for detection of antigen-antibody complexes immobilized on the PVDF membranes, according to the manufacturer’s protocol. After enhancement of the membranes by treatment with chemiluminescent reagent, images were acquired using a cooled CCD camera (LAS-3000 Mini; Fujifilm, Tokyo, Japan) and analyzed using image analysis software (MultiGauge; Fujifilm). A 40 kDa band MagicMark™ XP Western Protein Standard (Invitrogen) was used to determine protein size.

### Immunohistochemistry

Tissue samples were longitudinally incised and placed on the hard cardboard followed by fixing both ends of the samples with metal surgical clips to maintain the original length. The samples were then fixed in 4% paraformaldehyde/phosphate-buffered saline (pH7.0), embedded in paraffin, and sectioned at 4–5 μm thickness using a cryostat. All samples were stained with hematoxylin-eosin (HE), Masson’s trichrome and Victoria blue-van Gieson. The samples were then fixed in 4% paraformaldehyde/phosphate-buffered saline (pH7.0), embedded in paraffin and sectioned at 4–5 μm thickness using a cryostat. All samples were stained with hematoxylin-eosin (HE), Masson’s trichrome and Victoria blue-van Gieson. Immunostaining of the paraffin sections was performed with ASMA (1:1000; Dako), calponin (1:4000; Sigma), MHC (1:1000; Sigma), SM2 (1:1000; abcam) and SMemb (1:1000; Yamasa). All histological examinations and measurements were performed using a microscope (Biozero BZ-8000; Keyence, Osaka) and analysis software (BZ-Analyzer; Keyence).

### VSMC layer thicknesses and ratio

Masson’s trichrome stained Ao (n = 6), bPA (n = 6), mPA (n = 6) and IVC (n = 6) tissue samples were measured for total wall thickness and VSMC layer on 5–6 randomized points for each sample. The ratio of smooth muscle/wall thickness was calculated from the numerical values.

### Statistical analysis

The statistical significance of the findings was assessed using the Mann–Whitney *U*-test. All data are shown as means ± SEM, and *P* values less than 0.05 were considered to indicate statistically significant differences. IBM SPSS Statistics for Windows version 19.0 J (IBM Japan Ltd., Tokyo, Japan) was used for statistical analyses.

## Ethics

The study was approved by the Animal Care and Use Committee of Tokyo Women’s Medical University.

## Abbreviations

VSMC: Vascular smooth muscle cell; ASMA: Alpha smooth muscle actin; MHC: Myosin heavy chain; SMemb: Non-muscle myosin heavy chain B; Ao: Aorta; bPA: Branch pulmonary artery; mPA: Main pulmonary artery; IVC: Inferior vena cava; SEM: Standard error of the mean.

## Authors’ contributions

NI carried out all experimental studies and drafted the manuscript. GM participated in the design of the study and performed the statistical analysis and critically revised the manuscript for important intellectual content. KY provided project management support. All authors read and approved the final manuscript.

## Competing of interests

The authors declare that they have no competing interests.
